# Uncovering the sources of DNA found on the Turin Shroud

**DOI:** 10.1038/srep14484

**Published:** 2015-10-05

**Authors:** Gianni Barcaccia, Giulio Galla, Alessandro Achilli, Anna Olivieri, Antonio Torroni

**Affiliations:** 1 Laboratorio di Genomica, DAFNAE – Università di Padova, Via Università 16, 35020 Legnaro, Italy; 2 Dipartimento di Chimica, Biologia e Biotecnologie, Università di Perugia, Via Elce di Sotto 8, 06123 Perugia, Italy; 3 Dipartimento di Biologia e Biotecnologie “L. Spallanzani”, Università di Pavia, Via Ferrata 9, 27100 Pavia, Italy

## Abstract

The Turin Shroud is traditionally considered to be the burial cloth in which the body of Jesus Christ was wrapped after his death approximately 2000 years ago. Here, we report the main findings from the analysis of genomic DNA extracted from dust particles vacuumed from parts of the body image and the lateral edge used for radiocarbon dating. Several plant taxa native to the Mediterranean area were identified as well as species with a primary center of origin in Asia, the Middle East or the Americas but introduced in a historical interval later than the Medieval period. Regarding human mitogenome lineages, our analyses detected sequences from multiple subjects of different ethnic origins, which clustered into a number of Western Eurasian haplogroups, including some known to be typical of Western Europe, the Near East, the Arabian Peninsula and the Indian sub-continent. Such diversity does not exclude a Medieval origin in Europe but it would be also compatible with the historic path followed by the Turin Shroud during its presumed journey from the Near East. Furthermore, the results raise the possibility of an Indian manufacture of the linen cloth.

The Turin Shroud (TS) is a linen cloth, 4.4 m long and 1.1 m wide, bearing the double image of a man who suffered physical trauma in a manner consistent with crucifixion after being beaten, scourged and crowned with thorns^1^,^2^. TS is the most important relic of Christianity because the Catholic tradition identifies this burial cloth as that in which the body of Jesus Christ was wrapped before being placed in a Palestine tomb approximately 2000 years ago. Such a scenario is supported by numerous scholars who believe that the journey of TS began in Jerusalem in the year 30 or 33 AD^3^. After concealment for years, TS would have been first moved to Edessa (now Şanliurfa in Turkey) and then to Constantinople (now Istanbul in Turkey) in 944 AD. A burial cloth, which some historians consider the Shroud, was owned by the Byzantine emperors but disappeared during the Sack of Constantinople in 1204^4^. After this event, TS would have been taken by the crusaders and transferred to Athens (Greece), where it remained until 1225. Official documents attest that it was in France at Lirey around the years 1353 to 1357 and then was kept at Chambéry from 1502 to 1578, where passed into the hands of the Dukes of Savoy^3^,^4^,^5^. From 1578, apart from some brief displacements in an effort to hide it during war periods, TS was kept in Turin (Italy) and later placed in the royal chapel of the city Cathedral inside a specially designed shrine where it has been permanently conserved from 1694 to the present.

The TS shows many marks caused by human blood, fire, water and folding of the cloth that partially obscure the double, front and back, body image that is not yet reproducible^1^,^2^,^6^,^7^. In 1988, the age of the TS linen cloth was assessed by accelerator mass spectrometry. Results of radiocarbon measurements from distinct and independent laboratories yielded a calendar age range of 1260–1390 AD, with 95% confidence^8^, thus providing robust evidence for a Medieval recent origin of TS. However, two papers have highlighted some concerns about this determination^9^,^10^, and a Medieval age does not appear to be compatible with the production technology of the linen nor with the chemistry of fibers obtained directly from the main part of the cloth in 1978^1^,^11^.

In 1978 and 1988, dust particles were vacuumed from the interspace between the Shroud and the Holland Cloth sewn to it as reinforcement^12^. The composition of the particles was later studied in great detail by optical microscopy, and specimens from different filters were retained and characterized for their contents^6^,^13^. In past decades, pollen grains were classified to the genus and species levels using microscopy^14^,^15^,^16^, and the geographic areas where the corresponding plants originated and now inhabit proved to be compatible with the reported historic path followed by TS during the postulated 2000-year journey from the Near East^3^, thus supporting the authenticity of the relic.

In this study, we performed DNA analyses to define the biological sources of the dust particles (pollen grains, cell debris and other minuscule organic specimens, such as plant-derived fibers and blood-like clots) vacuum-collected in 1978 and 1988 in distinct TS filters, corresponding to the face, hands, glutei and feet of the body image^6^,^13^, and the lateral edge, which was used for radiocarbon dating^8^. To identify plant taxonomic entities and human genetic lineages, universal plant DNA sequences, including nuclear rDNA intergenic transcribed spacers (ITS) and chloroplast DNA (cpDNA) barcodes, and human mitochondrial DNA (mtDNA) target regions were amplified and sequenced. This allowed the identification of DNA sources from a wide range of plant species and human mitogenomes belonging to numerous haplogroups. The overall findings were then evaluated to determine whether the geographic areas of origin and distribution of detected plant cpDNA species and human mtDNA haplogroups might provide novel clues concerning the origin of the Turin Shroud.

## Results

### Detection of plant DNA from the Turin Shroud and identification of plant species

More than 100 PCR-derived amplicons were recovered from genic and intergenic target regions, 77 of which were successfully sequenced and attributed to a genus or species source (19 different plant taxa). Approximately half of the DNA sequences derived from the TS lateral edge samples (filter I) and allowed us to identify 16 plant species. In filters E-H, corresponding to various parts of the TS male body image, we assigned DNA sequences to one (glutei), two (feet), or three plant species (face and hands). Table 1 summarizes the plant species found on TS by querying databases of orthologous sequences from taxa of established identity. The land plant species include herbaceous weeds and crops, woody trees and shrubs; some are native to Mediterranean countries and are widespread in Central Europe, North Africa and the Middle East, whereas others have a center of origin in Eastern Asia and the Americas, and hence they were not yet present into Europe during the Medieval period (Supplementary Table S1).

**Figure 1:**
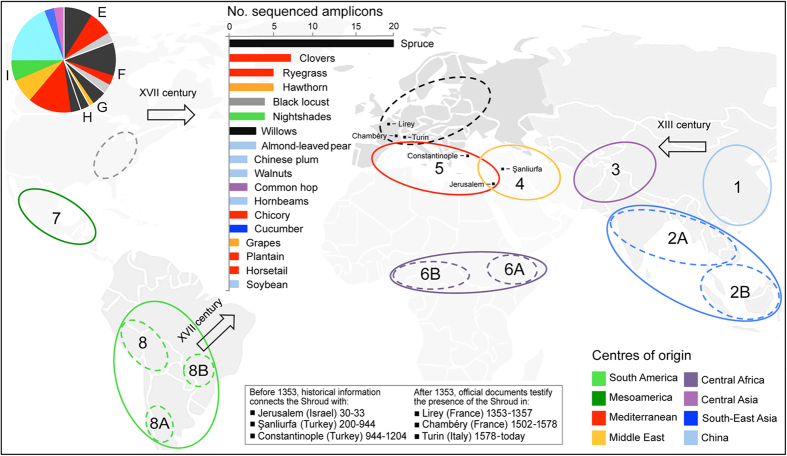
Plant DNA species found on the Turin Shroud. Schematic overview of the Vavilov centers of origin of plant taxa identified in TS samples. The number of amplicons is reported for each species and across filters E, F, G, H and I (see also Table 1 for details on the distribution of species among TS filters). The world map used as background to create this schematic overview has been obtained from Wikimedia Commons, the free media repository (https://commons.wikimedia.org/wiki/see file No_colonies_blank_world_map.png).

Among the taxa identified, the most abundant belongs to the genus *Picea*, which includes a few closely related species native to Europe (*P. abies* (L.) H. Karst., *P. obovata* Ledeb. and *P. omorika* Purk.) and several spruce trees widespread in temperate and boreal forest regions of the Northern Hemisphere. However, other species are present, including those native to the Mediterranean basin, such as clovers (*Trifolium* spp.), ryegrasses (*Lolium* spp.) and plantains (*Plantago* spp.), and Eastern Asia, such as uncommon forms of pear (*Pyrus kansuensis* Rehder) and plum (*Pyrus spinosa* Forssk, syn. *Pyrus amygdaliformis* Vill.) of the family Rosaceae. Among the plant species of the New World, black locust (*Robinia pseudoacacia* L.), a tree of the family Fabaceae native to Appalachia in the Eastern United States, is notable. In addition, we identified crop species largely grown by farmers and common in many agriculture systems of the Old World, including chicory (*Cichorium intybus* L.), common hop (*Humulus lupulus* L.), cucumber (*Cucumis sativus* L.) and grapevine (*Vitis* or *Parthenocissus* spp.). We also uncovered tree species commonly present in forests and woodlands, such as hornbeams (*Carpinus* spp.), walnuts (*Juglans* spp.) and willows (*Salix* spp.); although the centers of origin of these species are located in central Asia and Eastern Europe, their current areas of distribution are extremely broad (Fig. 1).

Based on the overall data, we may assume that TS was likely displayed in, or in very close contact with, different types of natural and anthropological environments. The large variety of taxonomic entities is compatible with highly diverse geographic ranges, varying from farms of cultivated plains to riparian woodlands and mountain forests. Some species have a center of origin and have diversified in areas around the Mediterranean basin, including North Africa and the Middle East, and most of these species were widely distributed throughout Europe before the age of Christ. However, other species identified on TS were not introduced to Europe before the XVI century, after the discovery of America by Christopher Columbus (for instance, *Robinia pseudoacacia* and also nightshades of the family Solanaceae), while the two species of the genera *Prunus* and *Pyrus*, rare fruit trees originating from South-East Asia and the Middle East, were likely introduced to Mediterranean territories from the XIII century and thereafter, following the travels of Marco Polo (Supplementary Table S1).

### Examination of human mtDNA haplotypes from the Turin Shroud and identification of mtDNA haplogroups

Among the 93 mtDNA amplicons generated and sequenced, a large number of different human sequences corresponding to three distinct mtDNA loci (MT-DLOOP, MT-CO1, MT-ND5) were identified. This result not only indicates that human DNA was indeed unequivocally present in the dust from TS but also that the sources of human DNA could be ascribed to numerous individuals (Table 2). In fact, the mtDNA haplotypes were found to belong to different branches of the human mtDNA tree (Supplementary Table S2), even after having excluded all the mtDNA sequences that could be theoretically attributed to operator contamination (Supplementary Table S3). Moreover, not only were the observed mtDNA haplotypes numerous, but they could also be affiliated to many distinct haplogroups. Six sub-branches of haplogroup H (H1, H2, H3, H4, H13, H33) are included, as well as representatives of haplogroups U2, U5, R0a, R7, R8, L3c, M39 and M56 (Table 2).

**Figure 2:**
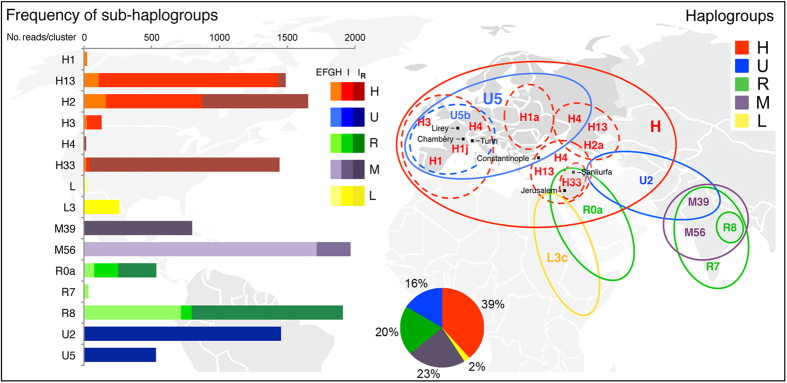
Human mtDNA haplogroups found on the Turin Shroud. Schematic overview of the current geographical distribution of human mtDNA haplogroups and sub-haplogroups identified in TS samples. The number and proportion of reads derived from samples EFGH, I and I_R_ are shown on the left for each haplogroup. The world map used as background to create this schematic overview has been obtained from Wikimedia Commons, the free media repository (https://commons.wikimedia.org/wiki/see file No_colonies_blank_world_map.png).

The number and proportion of read clusters derived from the different TS samples for each of the identified haplogroups are shown in Fig. 2, together with a schematic overview of their current geographic distributions. Some haplogroups are widespread, while others are geographically and ethnically more localized (see Supplementary Table S2 for detailed information). For instance, haplogroup H1 is very common in Western Europe, with a frequency peak among Iberians (~25%) but also among the populations of Northwestern Africa, including the Berbers. Haplogroup H4 is instead present at low and rather similar frequencies in Western (Iberia ~3%) and Eastern Europe (~1%), the Caucasus (~3%) and the Near East (~1%). Haplogroup H33 is rare and mainly found thus far among the Druze, a minority population of Israel, Jordan, Lebanon, and Syria. Haplogroup U2 is found largely in South Asia (~5%), but one of its subsets (U2e) is present in Europe, with a frequency of ~1%. Haplogroup U5 harbors an average frequency of 7% in modern European populations, and its major sub-branches, U5a and U5b, are most common in Eastern and Western Europe, respectively. Haplogroup R0a is predominantly localized in the Arabian Peninsula and the Horn of Africa, with the highest frequency in southeast Yemen (approximately 30%), though it is also found at low frequencies all over Western Eurasia. Haplogroup L3c is extremely rare and only found in East Africa, while haplogroups M39, M56, R7 and R8 are typical of the Indian subcontinent, with the latter essentially present only in Eastern India.

In brief, mtDNA data indicate that numerous individuals have left traces of DNA on the TS. Moreover, their mtDNA sequences belong to haplogroups that are typical of different ethnic groups and/or different geographic regions, including not only Europe where official documents verify the presence of TS since 1353 AD but also North and East Africa, the Middle East and even India.

### Collection of non-plant and non-human DNA sequences

It is worth mentioning that among all generated and sequenced cpDNA and mtDNA amplicons (~200 overall), only a few non-plant and non-human sequences were detected (Supplementary Table S1). One of these sequences, although very short being 58 bp long, partially matched and produced the best alignment with the MT-CYB gene (accession no. AY827092.1) from the southern grey shrike (*Lanius meridionalis* Koenigi), a medium-sized passerine bird that is reported to be resident in Southern Europe, Northern Africa and the Near East. Another of these sequences, corresponding to 694 bp of the CO1 gene, was ascribable to a marine worm (*Cerebratulus longiceps* Coe), rather common in the Northern Pacific Ocean, next to Canada (accession no. JQ007428-JQ007431).

## Discussion

DNA extracted from dust particles that were vacuumed from the Turin Shroud shows sequence profiles that identify numerous plant species and correspond to several distinct human mtDNA haplogroups. These results not only confirm that plant fibers and pollen grains are present on TS, as previously reported by optical microscopy, but also reveal that multiple human individuals touched or otherwise left traces of their DNA on the relic linen. The detection of such a variety of DNA sources is extremely valuable in assessing whether there are possible parallelisms between the areas of origin and distribution of identified land plant species and human mtDNA haplogroups and the temporal and spatial paths associated with the two alternative scenarios that have been proposed to explain the TS origin.

The radiocarbon measurements would place the origin of the TS linen in the time frame 1260–1390 AD. This not only implies a Late Middle Age origin, but a geographical path for the TS that is essentially restricted to Western Europe. In this scenario, the DNA traces that we detected could have entered in contact with the TS only rather recently, at most in the last 800 years, and these biological sources (plants and human subjects) had to be present in the geographic areas (France and Italy) where the TS was located and/or displayed. The alternative scenario implies instead a much longer journey that started in Jerusalem in the year 30 or 33 AD. In this case, the time frame for the interaction with the DNA biological sources is much longer (2000 years) and the geographic areas where the TS was located include the Near East, Anatolia, Eastern and Western Europe, with a potentially much wider range of plant and human interactions.

With regard to the land plant species identified, some are native to Mediterranean countries and widespread throughout Europe, North Africa and the Middle East and are thus compatible with both a rather recent Medieval origin in Europe and a more ancient Near Eastern origin. However, others have a center of origin in Eastern Asia and the Americas and were introduced to Europe only after the Medieval period. Clearly, the latter species cannot help in discriminating between alternative scenarios.

The quantitatively most abundant species found on TS dust is spruce: the vast majority of DNA sequences assigned to the genus *Picea* are likely attributable to the species *Picea abies* (L.) H. Karst., a forest tree that typically occupies highland areas of the Carpathians and Alps. Among the NCBI sequences most similar to those obtained from TS, one belongs to a spruce tree sampled in the Swiss Alps (accession number AF327585); this discovery is in accord with the transport of TS through the French-Italian Alps in 1578 when the relic was moved from Chambéry to Turin. Of note, our trnL-intron sequences shared the haplotype with most of those of *P. abies* accessions deposited in public databases, supported by specimen vouchers and annotated with a Southern European origin (*e*.*g*., Serbia) and an Eastern Asian origin (*e*.*g*., China), revealing not only common SNPs but also many private polymorphisms (accession numbers JQ007384-JQ007406 and Supplementary Figure S1).

Most of the plant species identified based on cpDNA and ITS sequences (*e.g.*, clovers, ryegrasses, plantains and chicories) have an origin and are now widespread in regions around the Mediterranean basin, from the Iberian Peninsula to Palestine. The presence of some alien tree species introduced from Eastern USA (*Robinia pseudoacacia* L.), and Northern or Southern China (*Salix suchowensis* W.C. Cheng, *Pyrus spinosa* Forssk and *Prunus kansuensis* Rehder) is not negligible, with the former species that currently has a distribution area centered in Europe and the Mediterranean basin, and the latter that are more widespread in temperate Asia, Southern Europe and Northern Africa. Overall, the various plant species and numerous taxonomic families identified on TS (Supplementary Table S1) suggest that contamination may have occurred during the past centuries and are compatible with the scenario that the linen cloth was exposed to different locations across the Mediterranean area.

With regard to the sources of human DNA, the detected haplotypes do not cluster randomly on the entire human mtDNA tree, but only on a specific subset of its branches, corresponding to numerous haplogroups from Western Eurasia and surrounding areas (Supplementary Table S2). This finding not only indicates that many individuals have left traces of their DNA on the TS, but also that they most likely belonged to different ethnic groups and were from far away geographic regions, including Europe, North and East Africa, the Middle East and India. Thus, the sources of these sequences fit well the geographic path of the postulated long journey from the Near East, even if they are also fully compatible with the scenario that among the perhaps thousands of worshippers who came into contact with the relic in France and Italy throughout the centuries, there were many coming from the far away geographic areas where these mtDNA haplogroups are common. Moreover, it should be taken into account that filters E, F, G and H correspond to the internal parts of the linen cloth that enveloped the body of the TS man; in contrast, filter I derives from the lateral edge of the cloth. Because the cloth was folded, the lateral edges were not only in contact with the external environment much more than the internal and more protected body image but are also the TS regions that were handled the most. Thus, filters E, F, G and H might provide more reliable clues than filter I with regard to the TS earliest contacts and contamination and, therefore, its hypothesized historical path. There are six haplogroups (H4, L3c, M39, R7, U2 and U5) that were detected only in the dust particles from filter I. If these are excluded, four partially overlapping geographic regions are generally outlined by the remaining haplogroups: (i) the Middle East with haplogroups H13, H33 and R0a; (ii) Southeast Europe and Turkey with haplogroups H1a, H2a and H13; (iii) Western Europe, including France and Italy, with haplogroups H1j and H3; and (iv) India with haplogroups M56 and R8.

In conclusion, our results on human mtDNA traces detected on the TS are compatible with both alternative scenarios that i) the cloth had a Medieval origin in Western Europe where people from different geographic regions and ethnic affiliations came in contact with it, possibly moved by the worship for the Christian relic; ii) the linen cloth had a Middle Eastern origin and was moved itself across the Mediterranean area, consequently coming across a wide range of local folks and devotes in a longer time span. Even in the latter case (*i*.*e*., Jerusalem in Israel until approximately 500 A.D., Şanliurfa in Turkey until 944, Constantinople in Turkey until 1204, Lirey and Chambery in France from 1353 until 1578, Turin in Italy to date)^3^, the detection of mtDNA haplogroups that are typically from India is somehow unexpected. One obvious possibility is that during the course of centuries, individuals of Indian ancestry came into contact with TS. Taking into account the rate of DNA degradation and PCR-biases toward undamaged DNA, the recent contamination scenario is extremely likely. However, one alternative and intriguing possibility is that the linen cloth was weaved in India, as supported perhaps by the original name of TS - *Sindon* - which appears to derive from *Sindia* or *Sindien*, a fabric coming from India.

## Methods

### Collection of TS Samples for DNA analyses

The samples used in this study for DNA investigations are sub-sets of the dust particles vacuumed from the back of the Turin Shroud (TS), which were kindly provided in 2010 by Giulio Fanti (Dept. of Industrial Engineering, University of Padua, Italy), who in turn received them personally from Giovanni Riggi di Numana in 2006^12^,^18^ (see also Supplementary Document S1). In particular, four samples were originally collected on filters in 1978 from four distinct areas of the back of TS, corresponding to the hands (filter E), face (filter F), feet (filter G) and glutei (filter H) of the TS male image^12^. An additional sample was collected in 1988, deriving specifically from the corner area (filter I) of TS, which was used for radiocarbon dating^8^,^12^ (for details, see Supplementary Figure S2, panels A–C).

The dust samples collected on these filters were immobilized on adhesive tapes as previously described^6^,^13^ and consisted of a variety of particles. In fact, optical microscope observations of filters E-I had previously revealed the presence of pollen grains, cell debris and other minuscule organic specimens, such as plant-derived fibers and blood-like clots^6^,^7^,^13^.

The five pieces of adhesive tape containing TS dust particles collected on filters E, F, G, H and I were supplied as portions of the original samples (ranging in size from 5 × 10 mm to 10 × 30 mm, see the schematic representation in Supplementary Figure S2, panel D), each attached to a sterile microscope slide. Each piece of adhesive tape was manually cut using a scalpel and tweezers under a stereomicroscope into tiny sub-portions of dimensions on the order of a few square millimeters (~5 × 5 mm each); the pieces were then individually transferred into 1.5-ml sterile microtubes. A total of 2 to 12 square portioned specimens of the pieces of adhesive tape were used for independent genomic DNA extractions; the DNA samples from the individual specimens were kept separate for PCR amplifications.

PCR amplicons obtained from each of the cpDNA regions were sub-cloned and used individually for DNA sequencing, whereas PCR amplicons obtained from each of the mtDNA regions were pooled for 454 sequencing reactions. In particular, the DNA amplicons from each of the 2, 4 or 6 specimens associated with internal filters E, F, G and H were pooled together into single samples, whereas the DNA amplicons from external filter I were divided into two samples of 6 specimens each (named I and I_R_ and taken as independent biological replications; Supplementary Figure S2). This pooling strategy was imposed by the different sizes of the pieces of adhesive tape to be analyzed for each of the five TS filters and by the fact that dust particles were demonstrated to be much more abundant on the tape representing the external filter than on those for the internal filters^6^,^13^.

All manipulation steps were performed aseptically under laminar flow hood conditions using autoclaved disposables and buffers that were filter-sterilized. In particular, we used sterile materials and all standard procedures with internal negative controls to avoid, or eventually discover, contaminations from the operators and the environment. The entire mitochondrial genomes of the three operators who came in contact with the Shroud samples were completely sequenced and all mtDNA sequences that we obtained from the TS samples and could be theoretically attributed to operator contamination (Supplementary Table S3) were excluded from our final results. As for possible environmental contaminations, none of the large varieties of plant sources detected in the Shroud (Supplementary Table S1) through the analysis of nuclear ITS, chloroplast genes or cpDNA sequences are grown or studied in our facilities.

### Genomic DNA extraction 

Specimens from the pieces of adhesive tape with the TS dust particles were used individually for genomic DNA extraction to avoid any loss of TS material and to perform replicate experiments for each TS filter.

All genomic DNA extractions were performed with the QIAamp^®^DNA Investigator commercial kit (Qiagen) by applying the protocol originally developed for laser-microdissected specimens, with some modifications. All manipulation steps were performed under laminar flow hood conditions using DNA- and DNase-free, disposable autoclaved materials and filter-sterilized buffers. Immediately after collection, sub-samples were transferred to a 1.5-ml tube containing a pre-warmed mixture composed of 40 μl of ATL buffer and 20 μl proteinase K. Each sample was then mixed by pulse-vortexing and incubated at 56 °C for 16 hours under constant agitation. After this step, 100 μl of the AL buffer, containing 2 μg of carrier RNA, was added, and the solution was mixed by pulse-vortexing for 15 sec. Then, 100 μl absolute ethanol was added, and the solution was mixed thoroughly by pulse-vortexing for 15 sec and then incubated for 5 min at room temperature. The next steps, which consisted of DNA immobilization to a silica membrane and wash steps to clean the isolated DNA from salts and impurities, were conducted by following the manufacturer’s suggestions. Elution of the DNA immobilized by the silica membrane was performed in two steps by using 15 μl ATE buffer at each elution step and by extending the incubation step to 10 min at room temperature.

To identify human genetic lineages and plant taxonomic entities, specific plant DNA and human mitochondrial DNA regions were designated for PCR amplification and sequencing.

### PCR amplification and sequencing of plant and human DNA sequences

PCR amplifications of plant DNA sequences were performed with primer combinations designed using nuclear (rDNA intergenic transcribed spacers, ITS) and chloroplast targets (cpDNA barcodes RuBisCO or rbcL, trnH-psbA, and trnL-intron) (Supplementary Table S4), following already available protocols^19^,^20^. Briefly, the reactions were performed in a total volume of 20 μl that included 2 μl of 10X reaction buffer, 1 mM MgSO_4_, 0.3 mM dNTPs, 0.25 U of Platinum^®^ *Pfx* DNA Polymerase (Life Technologies), 0.3 μM of primer mix and 2.5 μl of eluted DNA solution. The reactions were performed in a 9700 Thermal Cycler (Applied Biosystems) using a temperature profile that consisted of an initial denaturation step of 10 min at 95 °C followed by 50 cycles of 1 min at 95 °C, 30 sec at 55 °C, 1 min at 68 °C and a final step of 10 min at 68 °C.

The same conditions were adopted for PCR amplifications of human sequences corresponding to three distinct mtDNA loci (MT-DLOOP with the hypervariable segments MT-HV1 and MT-HV2, MT-CO1, and MT-ND5) using specific primer pairs (Supplementary Table S4).

The amplicons were sub-cloned by ligation into the TOPO-blunt cloning vector (Life Technologies) and transformed into chemically competent one-shot TOP10 bacterial cells (Life Technologies). Clones were plated on LB plates (1.5% agar, 50 μg/mL ampicillin, 40 μg/ml X-Gal), and transformed colonies were selected by Colony-PCR. Amplification reactions were performed in a total volume of 20 μl including 2 μl of 10X reaction buffer, 1.5 mM MgCl_2_, 300 μM dNTPs, 1.5 U of BIO*Taq* DNA polymerase (BIOLINE), 0.2 μM of M13For (5′-GTAAAACGACGGCCAG-3′) and M13Rev (5′-CAGGAAACAGCTATGAC-3′) primers. Positive colonies were sequenced using an ABI3100 automated sequencer (Applied Biosystems).

Both nuclear ITS sequences and DNA barcodes were used to identify plant and fungal species^21^,^22^, querying the Barcode of Life Data BOLD Systems v. 3 (http://www.boldsystems.org/index.php/IDS_OpenIdEngine). Sequence similarity searches in nucleotide collections (nt) and with non-redundant protein sequences (nr) were also performed with blastn/x programs of the Basic Local Alignment Search Tool BLAST v. 2.2.30+ (http://blast.st-va.ncbi.nlm.nih.gov/Blast.cgi) using default parameters. All plant cpDNA and ITS sequences deriving from PCR amplicons were verified through replicated sequencing of both strands and deposited in GenBank with accession numbers JQ007354-JQ007431 and JQ082521-JQ082524. The human mtDNA sequences, as clusters deriving from the assembly of reads generated by 454 sequencing, were deposited in GenBank with accession numbers KP126143-KP126230.

For each plant sequence, we collected information regarding: i) the TS location source; ii) the GenBank accession number of the most similar sequence; iii) the Linnaean name of the species, including the botanical family and its common name; iv) the name and length of the target DNA sequence; v) the center of origin and geographical distribution of the species, along with information on plant type and/or common use (for details, see Supplementary Table S1). From an experimental point of view, it should be noted that some species were identified in biological replicates by using different specimens of the same filter as well as different filters but were also assessed by technical replicates according to amplicons from different genetic targets. In addition, other species were identified by performing independent experiments, meaning that the PCR analyses were conducted at different times using DNA samples isolated at different times from the same filters/dust samples. All these cases are pivotal experimental validations and demonstrate the robust assignments of plant species (or genus).

### PCR amplification of human mtDNA and preparation of libraries for pyrosequencing

Amplifications of target mtDNA sequences prior to pyrosequencing were performed as described in the previous paragraph using specific primer sets (Supplementary Table S4). It is worth mentioning that the successful amplification of human mtDNA fragments ranging in size from 419 to 576 bp (as well as plant nuclear and chloroplast DNAs from 229 to 622 bp) such as the ones that we amplified, would be mostly possible from undamaged or slightly damaged DNA templates, which is unexpected when dealing with very ancient DNA specimens. However, the size of ancient DNA fragments, in addition to age, is influenced by many additional factors such as environmental conditions (*e*.*g*., temperature, moisture and pH) and mode of preservation (*e*.*g*., museum specimens *vs.* freshly excavated remains). In brief, DNA degradation becomes more effective only over long intervals, but is *de facto* unpredictable over shorter time spans such as those postulated for the DNA traces on the TS and when considering the numerous copies of extra-nuclear DNA molecules^23^,^24^,^25^,^26^.

PCR products originating from the amplification of multiple samples were pooled (Supplementary Table S4) and purified with QIAquick PCR Purification Kit (QIAGEN) by following the recommendations of the supplier. Next, the amplicons of the three different mtDNA target regions were pooled together, as reported in Supplementary Table S5, and purified with the Agencourt AMPure XP procedure (Beckman Coulter) by following the recommendations of the supplier. Quantification of the pooled amplicons was performed with a Nanodrop fluorometer ND 3300 (Thermo Scientific).

Libraries were prepared starting from 100 ng of purified PCR products using Rapid Library Preparation Method (Series GS FLX+; Roche 454 sequencing), starting from step 3.2: Fragment End Repair (thus avoiding the step 3.1, DNA Fragmentation by Nebulization). Single libraries were tagged with univocal MIDs (Supplementary Table S6). Emulsion PCR and 454 sequencing were carried out according to the manufacturer’s instructions on the Roche 454 FLX Titanium platform. Library sequencing was carried out using a Roche 454 GS Junior System.

### Analysis of sequence reads, variant calling and cluster assembly

Sequence reads were de-multiplexed based on their MID sequence with the Roche 454 software SFFfile. The number of sequenced and aligned reads referring to the single libraries are reported in Supplementary Table S7.

Mapping of the sequence reads to the *H. sapiens* mitochondrial genome (gb|NC_012920.1) was performed with the software CLC Genomics Workbench version 5.05 with defaults parameters. Mapping of the sequence reads originating from each library was used to estimate the average coverage for each target region. Background noise in the sequences was filtered with a program developed ad hoc that analyzes the composition in words (k-mer) of the produced sequences and masks the words having a frequency lower than the value set as the cutoff. For this step, the length of the k-mers was set to 7, and the minimum frequency parameter was set to 5. After the masking of rare variants, the sequences were clustered with the software CD HIT v.4.5.4^27^ by setting a threshold of percent identity equal to 99%. The average coverage of the mtDNA target regions for each single pool and target region is summarized in Supplementary Table S7.

Sequence clusters with a length greater than 200 bp were aligned to the *H. sapiens* mitochondrial genome (gb|NC_012920.1) using the program “bwa”^28^ with default parameters. In this step, each pool of sequences was independently aligned against the reference sequence^17^. The alignment files were saved as standard SAM files. Variant calling was performed on alignments of sequences representative of each cluster with the software PASS^29^. For each cluster, the haplotype was defined as the collection of substitutions (either transversions or transitions) that emerged in the variant calling process. Insertions and deletions were not taken into account.

A pre-filtering step for estimation of the mtDNA haplogroups represented in our cluster sequences was performed with the software HaploGrep^30^, which allows the automatic assignment of haplogroups according to Phylotree^31^. Preliminary assignments of haplogroups to sequence clusters displaying the highest sequence coverage were manually verified. The frequency of each haplogroup was estimated by the number of sequences contained in each cluster assigned to that specific haplogroup. The distribution of haplogroups in the different samples was estimated as above, but by counting the sequences according to the library information. The assembled human mtDNA sequences were deposited in GenBank with accession numbers KM655881-KM655934 (for details, see Supplementary Table S8).

## Additional Information

**How to cite this article**: Barcaccia, G. *et al.* Uncovering the sources of DNA found on the Turin Shroud. *Sci. Rep.* **5**, 14484; doi: 10.1038/srep14484 (2015).

## Supplementary Material

Supplementary Information

## Figures and Tables

**Table 1:** Plant DNA species found on the Turin Shroud.

**GenBank Accession No.**	**TS Filters**	**Genus/Species**	**Taxonomy ID**	**Common Name**	**Gene/Barcode**	**Sequence length (bp)**	**Best Match Accession No.**	**E-value**	**Identity (%)**
JQ007384−JQ007389	E	*Picea* spp.^2^	3328	Spruce	trnL	563	HF937082.1^7^	0e + 00	99–100
JQ007391/JQ007393−JQ007398	F	*Picea* spp.^2^	3328	Spruce	trnL	563	HF937082.1^7^	0e + 00	99–100
JQ007399−JQ007400	G	*Picea* spp.^2^	3328	Spruce	trnL	563	HF937082.1^7^	0e + 00	99–100
JQ007402	G	*Picea* spp.^2^	3328	Spruce	trnL	315	JX508522.1^7^	4e–161	99
JQ007403−JQ007404	H	*Picea* spp.^2^	3328	Spruce	trnL	516−518	HF937082.1^7^	0e + 00	99
JQ007405−JQ007406	I	*Picea* spp.^2^	3328	Spruce	trnL	563	EF440555.1^7^	0e + 00	99–100
JQ007355/JQ007413	E	*Robinia pseudoacacia* L.^3^	35938	Black locust	ITS	344–348	DQ006010.1	9e–163	98–99
JQ007359/JQ007360	F	*Robinia pseudoacacia* L.^3^	35938	Black locust	ITS	344	DQ006010.1	5e–170	99
JQ007407/JQ007410	I	*Trifolium fragiferum* L.	97023	Strawberry clover	trnL	610	DQ311791.1	0e + 00	99–100
JQ007361−JQ007363	I	*Trifolium striatum* L.	3898	Knotted clover	ITS	412−417	AF053172.1^7^	0e + 00	99
JQ007408−JQ007409	I	*Trifolium repens* L.	3899	White clover	trnL	621−622	JN617179.1	0e + 00	99
JQ007412	I	*Carpinus* spp.	12989	Hornbeams	trnL	530	AY211425.1^7^	0e + 00	99
JQ007381	I	*Carpinus* spp.	12989	Hornbeams	rbcL	256	KF418948.1^7^	5e–130	100
JQ007390/JQ007392	F	*Cichorium intybus* L.	13427	Chicory	trnL	518	GU817987.1	0e + 00	100
JQ007372/JQ007373	I	*Cucumis* spp.^4^	3655	Cucumber	trnH−psbA	275−276	KF957866.1^7^	1e–140	99–100
JQ007368/JQ007369	I	*Humulus lupulus* L.	3486	Common hop	trnH−psbA	414	FN687524.1	0e + 00	99
JQ007382/JQ007383	I	*Juglans regia* L.	16718	Persian walnut	trnL	599−600	AY231167.1	0e + 00	100
JQ007354/JQ007356^1^	E	*Lolium multiflorum* Lam.^5^	4521	Annual ryegrass	ITS	333	X99974.1	0e–150	96
JQ007357/JQ007358	E	*Lolium multiflorum* Lam.^5^	4521	Annual ryegrass	ITS	329	X99974.1	4e–161	98
JQ007377	I	*Nicotiana* spp.	4085	Tobacco	rbcL	511	KM025249.1^7^	0e + 00	99
JQ007376^1^	I	*Solanaceae* fam.	4107	Nightshades	rbcL	234	KM025249.1^7^	4e–116	99
JQ007364/JQ007375	I	*Prunus kansuensis* Rehder	329890	Chinese plum	trnH−psbA	388	KF990036.1	0e + 00	99
JQ007370	I	*Pyrus spinosa* Forssk^6^	1143245	Almond-leaved pear	trnH−psbA	229	HG737342.1	3e–103	97
JQ007371/JQ007374	I	*Pyrus spinosa* Forssk^6^	1143245	Almond-leaved pear	trnH−psbA	382−383	HG737342.1	0e + 00	98–99
JQ007365−JQ007367	I	*Salix suchowensis* W.C. Cheng	1278906	Shrub willow	trnH−psbA	344−345	KM983390.1	8e–174	99
JQ007379	I	*Equisetum* spp.	3257	Horsetail	rbcL	461	AB574684.1^7^	0e + 00	100
JQ007380	I	*Glycine max* (L.) Merr.	1462605	Soybean	rbcL	469	KF611800.1^7^	0e + 00	99
JQ007378^1^	I	*Crataegus* spp.	23159	Hawthorn	rbcL	477	KJ506869.1^7^	0e + 00	100
JQ007411	I	*Plantago argentea* Chaix	185776	Plantain	trnL	582	AJ430931.1	0e + 00	99
JQ007401	G	*Vitis* or *Parthenocissus* spp.	3603 or 3606	Grape	trnL	594	AB235078.1^7^	0e + 00	99

Plant cpDNA and ITS sequences detected in TS samples and their putative species or genus source (verified on 7 March 2015 using blastn/x programs of BLAST v. 2.2.30+ of the NCBI nr nucleotide database and BOLD Systems v. 3 for rbcL and ITS databases).

^1^Accessions associated to distinct PCR amplicons (from 2 to 5) sharing the same DNA sequence.

^2^Different *Picea* species, including *P. abies* (L.) H. Karst., scored the same E-values in the NCBI database of nucleotide sequences (for details on informative SNPs, see also Supplementary Figure S1).

^3^Analysis of the BOLD using the ITS sequences as query revealed similarity with *Glycyrrhiza glabra* L. (Licorice) accessions but with less robust E-values.

^4^These *Cucumis* cpDNA sequences scored the same E-values with *Cucumis sativus* L. (cultivated cucumber) and *Cucumis hystrix* Chakr. (wild cucumber) accessions of the NCBI database.

^5^*Lolium perenne* L. and *Festulolium holmbergii* (Dörfl.) P. Fourn. (*Festuca arundinacea* x *Lolium perenne*) are also possible according to ITS nucleotide sequence similarities found in the BOLD.

^6^Synonymous name *Pyrus amygdaliformis* Vill.

^7^Multiple accessions of the same species with equivalent E-values in the NCBI database of nucleotide sequences.

**Table 2:** Human mtDNA haplogroups found on the Turin Shroud.

**GenBank Accession No.**	**TS Sources**	**MT Locus**	**Sequence Range (from np to np)**	**Haplotype**^1^,^2^,^3^	**Haplogroup**	**Sub-Haplogroup**	**Quality**^4^
KM655914	EFGH	DLOOP	16303–16569;1–154	16519C, 73G	H1	H1a	100
KM655923	EFGH	HV1	15986–16425	16240G	H1	H1j8/H1bz	100
KM655924	I	HV1	15976–6419	16234T	H13	H13a1d	100
KM655908	EFGH	CO1	6977–7492	7173G, 7403G, 7404A	H13	H13b1b	100
KM655909	I_R_	CO1	6977–7494	7403G, 7404A	H13	H13b1b	100
KM655926	I_R_	HV2	16–428	263G, 286T	H2	H2a2a	100
KM655932	EFGH	HV2	10–428	263G, 363C	H2	H2a2a	100
KM655928	EFGH	HV2	10–426	263G, 410T	H2	H2a2a	100
KM655881	I_R_	ND5(−5P)	12279–12763	12385A, 12418T, 12427A	H2	H2a2a1	0
KM655910	I_R_	DLOOP	16285–16569;1–154	16375A, 16419A, 16519C	H2	H2a2a1	0
KM655903	I_R_	CO1	6977–7552	7316A	H2	H2a2a1	0
KM655904	I	CO1	6977–7494	7342T, 7402G	H2	H2a2a1	0
KM655902	I_R_	CO1	7093–7472	7402G	H2	H2a2a1	0
KM655889	I	ND5(−5P)	12279–12763	12399T, 12441C	H3	H3ae	100
KM655883	EFGH	ND5(−5P)	12279–12763	12314C, 12372A, 12419T	H3	H3au	100
KM655918	EFGH	HV1	15980–16425	16188T	H33	H33c	100
KM655917	I_R_	HV1	15976–16425	16188T	H33	H33c	100
KM655922	I_R_	HV1	15976–16425	16188T	H33	H33c	100
KM655916	I	HV1	15980–16425	16188T	H33	H33c	100
KM655915	I_R_	HV1	15976–16425	16188T	H33	H33c	100
KM655921	I_R_	HV1	15978–16425	16179A	H4	H4a1c2	100
KM655907	EFGH	CO1	6997–7495	7028N^1^, 7132T, 7146G, 7232T, 7256T, 7316A	L	L2'3'4'5'6	77
KM655906	I	CO1	7051–7532	7028N^1^, 7394G	L3	L3c	73
KM655913	I_R_	DLOOP	16286–16569;1–154	16519C, 66T	M	M39	59
KM655901	I	ND5(−3P)	13320–13807	13753C	M	M56	100
KM655900	EFGH	ND5(−3P)	13320–13807	13753C	M	M56	100
KM655882	I	ND5(−5P)	12279–12763	12406A, 12738C	R7	R7a1	68
KM655896	I_R_	ND5(−3P)	13320–13807	13630G, 13782T	R8	R8a1	75
KM655899	EFGH	ND5(−3P)	13320–13807	13751C, 13782T	R8	R8a1	100
KM655898	I_R_	ND5(−3P)	13320–13807	13758A, 13782T	R8	R8a1	100
KM655897	I	ND5(−3P)	13320–13807	13767G, 13782T	R8	R8a1	100
KM655905	EFGH	CO1	6977–7552	7232T, 7256T, 7316A	R0a	R0a2e	72
KM655925	I_R_	HV2	14–428	58C, 60T^2^, 64T, 263G	R0a	R0a’b	100
KM655934	I	HV2	16–428	58C, 60T^2^, 64T, 263G	R0a	R0a’b	100
KM655933	EFGH	HV2	22–428	58C,60T^2^, 64T, 263G	R0a	R0a’b	100
KM655920	I_R_	HV1	15976–16425	16051N^1^, 16209N^1^, 16239N^1^, 16352C, 16353T	U2	U2b2	77
KM655895	I_R_	ND5(−3P)	13320–13807	13630G,13789C	U2	U2d2	73
KM655894	I_R_	ND5(−3P)	13320–13807	13617N^1^, 13630G, 13637N^1^	U5	U5b2b	69

Human mtDNA haplotypes and predicted haplogroups and sub-haplogroups detected in TS samples.

^1^N indicates that the specified diagnostic mutation falls in a low quality region.

^2^The diagnostic insertion 60+T is likely present as well.

^3^Mutations are relative to the rCRS^17^.

^4^According to HaploGrep (http://haplogrep.uibk.ac.at/).
