# Author Correction: Uncovering the sources of DNA found on the Turin Shroud

**DOI:** 10.1038/s41598-021-85973-1

**Published:** 2021-03-22

**Authors:** Gianni Barcaccia, Giulio Galla, Alessandro Achilli, Anna Olivieri, Antonio Torroni

**Affiliations:** 1 Laboratorio di Genomica, DAFNAE – Università di Padova, Via Università 16, 35020 Legnaro, Italy; 2 Dipartimento di Chimica, Biologia e Biotecnologie, Università di Perugia, Via Elce di Sotto 8, 06123 Perugia, Italy; 3 Dipartimento di Biologia e Biotecnologie “L. Spallanzani”, Università di Pavia, Via Ferrata 9, 27100 Pavia, Italy

Correction to: *Scientific Reports* 10.1038/srep14484, published online 5 October 2015

The Supplementary Information file that accompanies this Article contains an error where the reference list was erroneously omitted.

The corrected Supplementary Information file is linked to this correction notice.

## Supplementary Information

Supplementary Information.

