# Supplementary material for: Uncovering the sources of DNA found on the Turin Shroud
Source: Sci Rep. 2015 Oct 5;5:14484. doi: 10.1038/srep14484 (PMC4593049; doi:10.1038/srep14484)
Supplement: Supplementary Information [file srep14484-s1.doc]

**Uncovering the sources of DNA found on the Turin Shroud**

Gianni Barcaccia^1^*, Giulio Galla^1^, Alessandro Achilli^2^, Anna Olivieri^3^, Antonio Torroni^3^

^1^Laboratorio di Genomica, DAFNAE – Università di Padova, Via Università 16, 3520 Legnaro, Italy; ^2^Dipartimento di Chimica, Biologia e Biotecnologie, Università di Perugia, Via Elce di Sotto 8, 06123 Perugia, Italy; ^3^Dipartimento di Biologia e Biotecnologie "L. Spallanzani", Università di Pavia, Via Ferrata 9, 27100 Pavia, Italy.

***Corresponding author. E-mail: gianni.barcaccia@unipd.it**

**Supplementary Information**


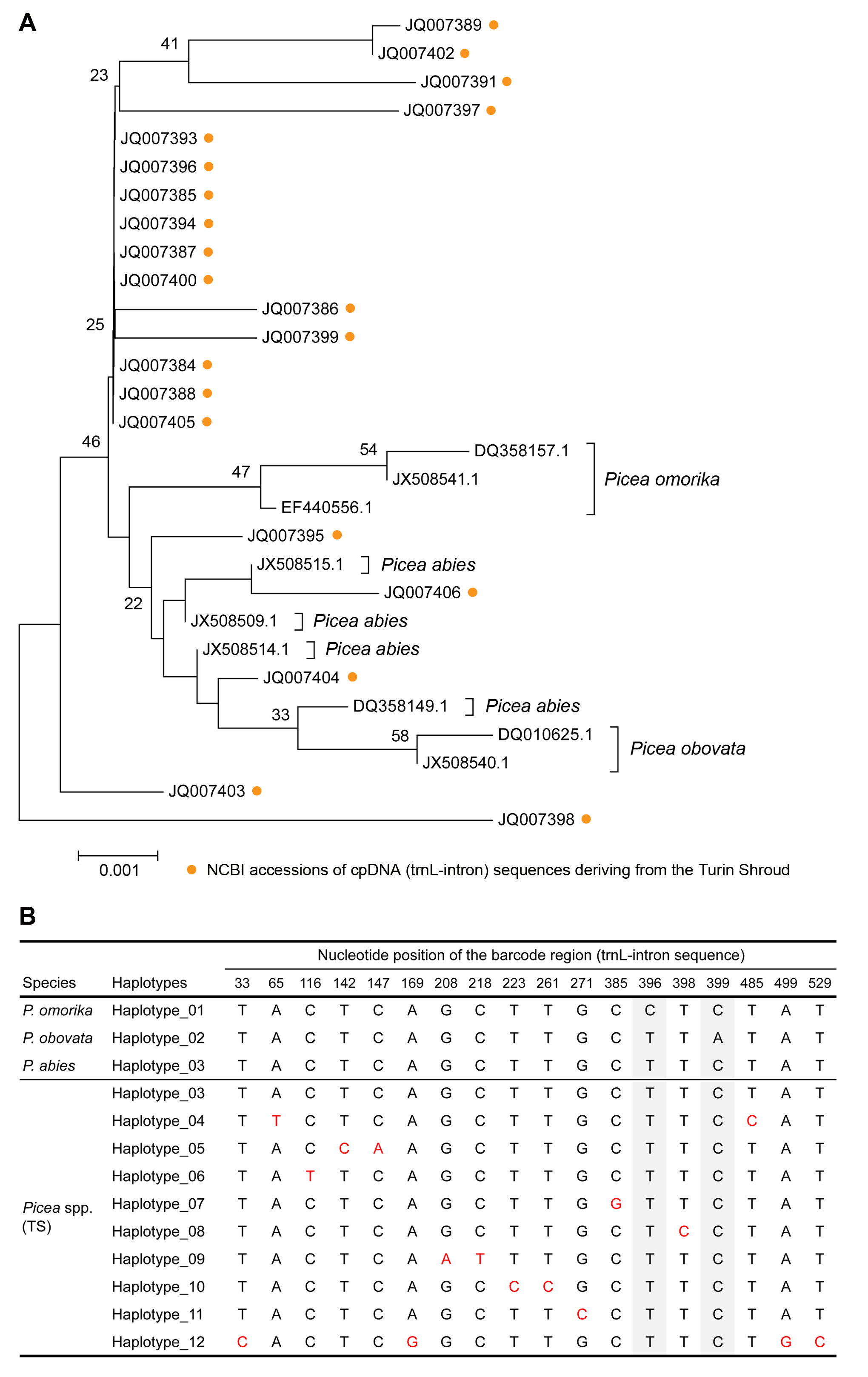


**Supplementary Figure S1.** **Analysis of the *Picea* tnrL-intron sequences using *P. abies* (L.) H. Karst. (Norway spruce), *P. obovata* Ledeb. (Siberian spruce) and *P. omorika* Purk. (Serbian spruce) accessions.** **Panel A:** Neighbor-Joining tree based on multiple alignment of the 20 trnL-intron sequences of *Picea* spp. deriving from the five filters E (6), F (7), G (3), H (2) and I (2) of the Turin Shroud (GenBank accession numbers JQ007384-JQ007406, see also Supplementary Table S1) together with 9 trnL-intron sequences of *P.* *omorika* (Haplotype_01: accession numbers DQ358157.1; EF440556.1; JX508541.1), *P. obovata* (Haplotype_02: accession numbers DQ010625.1; JX508540.1) and *P. abies* (Haplotype_03: accessions numbers DQ358149.1; JX508509.1; JX508514.1; JX508515.1) deposited in the NCBI databases and supported by specimen vouchers. A bootstrap analysis was conducted to measure the stability of the computed branches with 1,000 resampling replicates (bootstrap values higher than 20% are reported on the main nodes of the tree). **Panel B:** Sequence variation of the trnL intron of three closely related spruce species native to Europe, including *P. abies*, *P. obovata* and *P. omorika*. The trnL-intron sequences deriving from the TS revealed a few informative SNPs and many private variants. In particular, ten of these sequences shared the Haplotype_03 with *P. abies* accessions (accession numbers JQ007404; JQ007394; JQ007384; JQ007385; JQ007387; JQ007388; JQ007396; JQ007400; JQ007405; JQ007393), whereas the remaining sequences revealed nine distinct haplotypes (*i*.*e*., Haplotype_04: JQ007389; JQ007402; Haplotype 05: JQ007406; Haplotype_06: JQ007395; Haplotype_07: JQ007403; Haplotype_08: JQ007386; Haplotype_09: JQ007391; Haplotype_10: JQ007397; Haplotype_11: JQ007399; Haplotype_12: JQ007398).


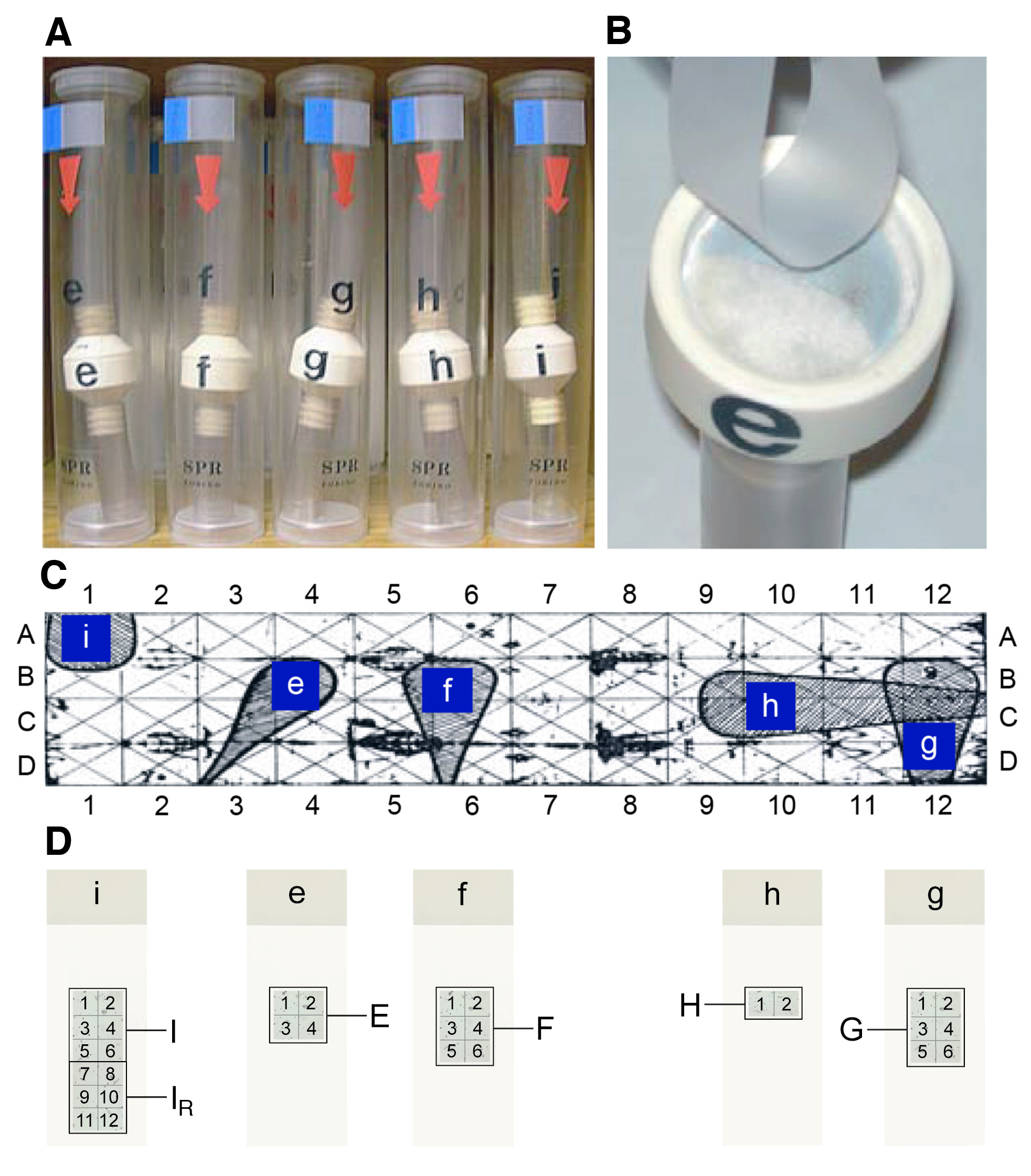


**Supplementary Figure S2. Turin Shroud (TS) samples used for DNA analyses.** **Panel A:** Samples of dust particles collected by Giovanni Riggi di Numana in 1978 from distinct areas of TS corresponding to four parts of the TS male body image (filters E-H) and in 1988 from the corner area of TS used for radiocarbon dating (filter I). **Panel B:** The dust samples collected on these filters were immobilized on pieces of adhesive tape and consisted of a variety of particles, including pollen grains, cell debris and other minuscule organic specimens, such as plant-derived fibers and blood-like clots. **Panel C:** Graphic chart of TS underling the four distinct areas of the back of TS, corresponding to the samples related to hands (filter E), face (filter F), feet (filter G) and glutei (filter H) of the body image, along with the additional sample (filter I) derived specifically from the corner area and used for radiocarbon dating. **Panel D:** Schematic representation of the five microscope slides mounted with the materials provided by Giulio Fanti in 2010, showing portions (ranging in size from 5 × 10 mm to 10 × 30 mm) of the original pieces of adhesive tape containing TS dust particles collected on filters E, F, G, H and I. Each of these pieces of adhesive tape was split into tiny sub-portions with dimensions of a few square millimeters (~5 × 5 mm each), which were then individually transferred to 1.5-ml sterile microtubes for DNA extraction.


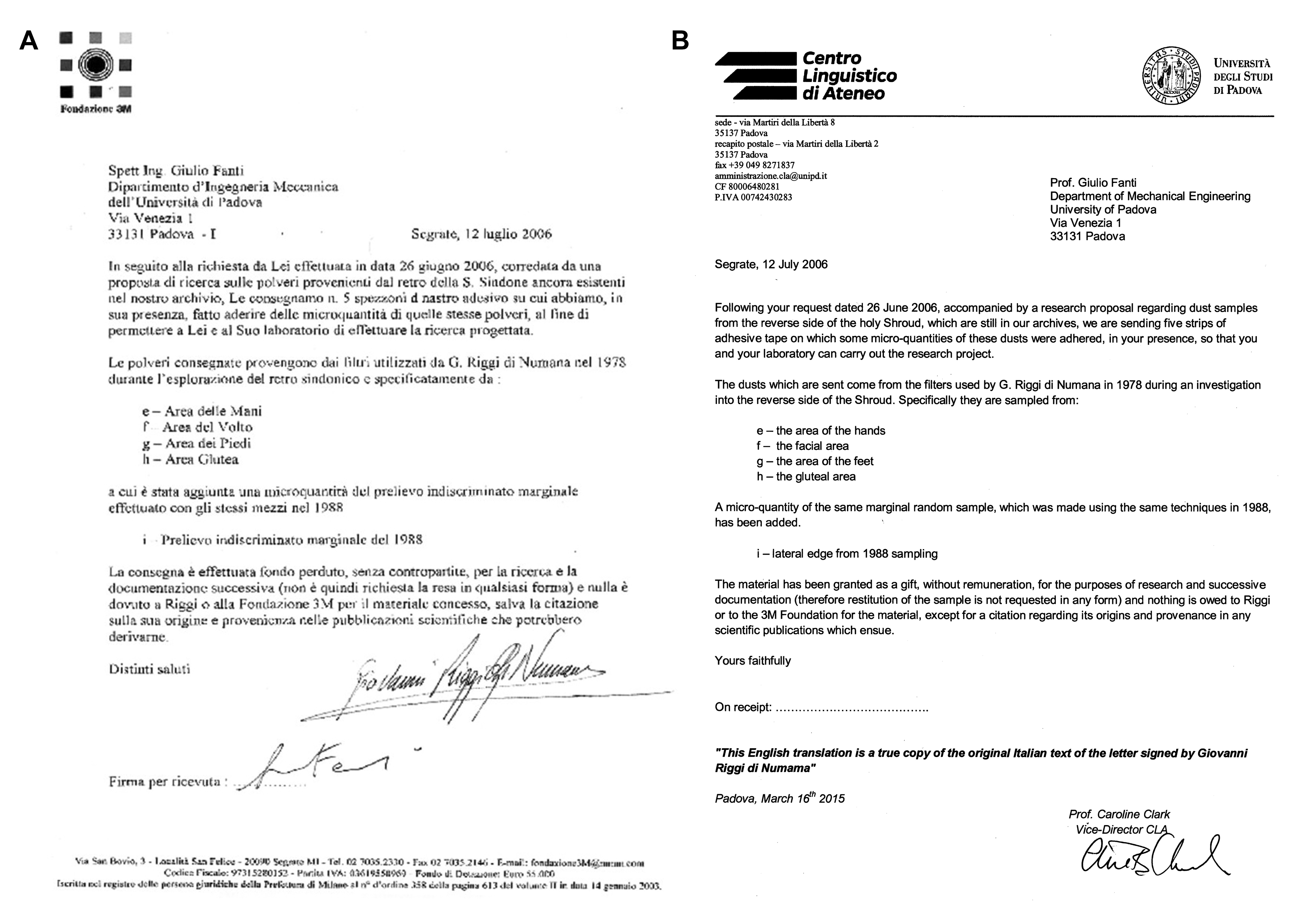


**Supplementary Document S1.** Original letter (in Italian) written by Giovanni Riggi di Numana to Giulio Fanti (**Panel A**); English translation of the original Italian text of the letter (**Panel B**). This letter, dated 12 July 2006, describes the five micro specimens of the Turin Shroud filters, deriving from the 1978 and 1988 samplings, which were sent from Giovanni Riggi di Numana, 3M Foundation of Milan, to Prof. Giulio Fanti, University of Padua, for the purposes of research^32^.

**Supplementary Table S1.** Plant cpDNA and ITS sequences along with non-human mtDNA sequences identified on the Turin Shroud and their putative species or genus source and accession number (verified on 7 March 2015 using blastn/x programs of BLAST v. 2.2.30+ of the NCBI nr nucleotide database and BOLD Systems v. 3 for rbcL and ITS databases).

^1^Different *Picea* species, including *P. abies* (L.) H. Karst. (Norway spruce) and *P. omorika* Purk. (Serbian spruce), are possibly identified (for details on taxonomic informative SNPs, see also Supplementary Figure S1).

(+)Multiple accessions with the same E-value in the NCBI database of nucleotide sequences.

Na, not applicable.

**Supplementary Table S2**. Current geographic distribution of human mtDNA haplogroups detected on the Turin Shroud.

| mtDNA haplogroup | Distribution range |
| --- | --- |
| **H1** | H1 encompasses an important fraction of Western European (13%) and Northwestern African mtDNAs (10%), reaching its frequency peaks among Northern Iberians (25%)^18–21^. Sub-haplogroup H1a is found especially in Eastern Europe, while H1j is mostly observed in Western Europe. |
| **H2a** | H2a includes the mtDNA reference sequence (rCRS) and is found at low frequency (2-3%) all over Western Eurasia with the highest frequencies in Eastern Europe and the Caucasus (5%)^19,22^. |
| **H3** | Its distribution partially overlaps that of H1 but with lower frequencies (3-4%). H3 is more frequent in Western (9%) than Eastern Europeans (2%), and it is extremely rare in the Near East^18–21^. |
| **H4** | It is present at low frequencies in Western (Iberia 3%) and Eastern Europe (1%), the Caucasus (3%) and the Near East (1%)^23^. |
| **H13** | Its distribution partially overlaps that of H4 but is mostly found around the Caucasus (2%) and the Near East (3%)^22^. |
| **H33** | Very rare haplogroup mainly found so far among the Druze^24^, a minority population of Israel, Jordan, Lebanon, and Syria. |
| **L** | Macro-haplogroup L is the most common all over sub-Saharan Africa and is also found at low frequencies in the Near East (2%) and Southern Europe (3%)^25^. |
| **L3** | Haplogroup L3 is mainly diffused in Africa, with frequency peaks (25%) in the Horn of Africa, while its derivative L3c is extremely rare and only found in East Africa^26^. |
| **M39 & M56** | Haplogroups M39 and M56 are mainly diffused in the Indian sub-continent^27,28^. |
| **R7** | Haplogroup R7 is distributed at low frequencies (<6%) in the Indian sub-continent^29^. |
| **R8** | R8 is essentially found only in Eastern India (frequency peak of 12%)^30^. |
| **R0a** | Haplogroup R0a is mainly localized in the Arabian Peninsula and the Horn of Africa, with the highest frequency in southeast Yemen (about 30%). It is also found at low frequencies all over western Eurasia^31^. |
| **U2** | Haplogroup U2 is found mostly in South Asia (5%)^32^. It is the rarest U sub-clade in the Middle East and Europe, where almost all U2 mtDNAs are members of U2e (1%)^33^. |
| **U5** | U5 is probably the most ancient European mtDNA haplogroup, with an average frequency of 7% in modern European populations. It is also found at lower frequencies in the Near East and North Africa^34^. U5a and U5b are most common in Eastern and Western Europe, respectively. |

**Supplementary Table S3.** Haplotype and haplogroup/sub-haplogroup classification of the complete mtDNAs from the three operators.

| **Sample ID** | **GenBank Accession No.** | **Complete mtDNA Haplotype^1^** | **Haplogroup** |
| --- | --- | --- | --- |
| Operator 1 | KP877126 | 263G, 309.1C, 315.1C, 750G, 1438G, 4769G, 6776C, 8860G, 9545G, 11863T, 12738C, 15326G, 16519C, | H3 |
| Operator 2 | KP877127 | 73G, 263G, 309.1C, 315.1C, 750G, 930A, 960.1C, 1438G, 2706G, 3197C, 4769G, 7028T, 8860G, 9477A, 9548A, 11467G, 11719A, 12308G, 12372A, 12399T, 12441C, 13617C, 14766T, 14793G, 15326G, 16192T, 16256T, 16266T, 16270T, 16526A | U5a2b1a |
| Operator 3 | KP877128 | 64T, 73G, 153G, 159C, 195C, 225A, 226C, 309.1C, 315.1C, 750G, 1438G, 1719A, 2706G, 4659A, 4769G, 6221C, 6371T, 7028T, 8860G, 9903C, 11204C, 11434T, 11719A, 12705T, 13966G, 14470C, 14766T, 15139C, 15326G, 15793T, 16092C, 16183C, 16189C, 16193.1C, 16223T, 16266T, 16278T, 16519C | X2n |

^1^Mutations are relative to the rCRS^17^. Suffixes indicate insertions of a C (.1C)

**Supplementary Table S4**. Primer pairs for PCR amplification of cpDNA and nuDNA sequences (plant species identification) and mtDNA sequences (human/animal identification).

| **Genome** | **Region/ Gene** | **Forward primer** | **Forward primer sequence** | **Reverse primer** | **Reverse primer sequence** | **Amplicon length (bp)** | **Reference** |
| --- | --- | --- | --- | --- | --- | --- | --- |
| **cpDNA** | trnH-psbA | psbA3'F | GTTATGCATGAACGTAATGCTC | trnHF | CGCGCATGGTGGATTCACAATCC | 342 | 37,38 |
|  | trnL | trnL-c | CGAAATCGGTAGACGCTACG | trnL-intron-rev | GGGGATAGAGGGACTTGAAC | 560 | 39 |
|  | rbcL | rbcL-for | GCAGCATTYCGAGTAASTCCBCA | rbcL-rev | GAAACGBTCTCTCCAWCGCATAAA | 436 | Present study |
| **nuDNA** | ITS | ITS5 | GGAAGTAAAAGTCGTAACAAGG | ITS2 | GCTGCGTTCTTCATCGATGC | 336 | 40 |
|  | ITS | ITS3 | GCATCGATGAAGAACGCAGC | ITS4 | TCCTCCGCTTATTGATATGC | 386 | 40 |
| **mtDNA** | CO1 | LepF1 | ATTCAACCAATCATAAAGATATTGG | LepR1 | TAAACTTCTGGATGTCCAAAAAATCA | 723 | 41 |
|  | CYTB | L14841 | CCATCCAACATCTCAGCATGATGAAA | H15149 | GCCCCTCAGAATGATATTTGTCCTCA | 360 | ^42^, shortened |
|  | CO1 | COI-LCO1490 | GGTCAACAAATCATAAAGATATTGG | COI-HC02198 | TAAACTTCAGGGTGACCAAAAAATCA | 708 | 43 |
|  | CO1 | COI454_for | AGCAAACTCATCACTAGACAT | COI454_rev | TTGACAAAGTTATGAAATGGTT | 515 | Present study |
|  | DLOOP | Dloop_for | CAAACCTACCCACCCTTAACAG | Dloop_rev | ATAGGATGAGGCAGGAATCAAA | 434 | Present study |
|  | DLOOP | L15400 | TCCACCATTAGCACCCAAAG | HVI_rev | GATATTGATTTCACGGAGGATGG | 445 | ^44^, Present study |
|  | DLOOP | HVII_for | TCTATCACCCTATTAACCACTCAC | H408 | TGTTAAAAGTGCATACCGCCA | 414 | ^45^, Present study |
|  | ND5 | ND53P_for | CATCTGTACCCACGCCTTCTT | ND53P_rev | GGGCTGTGAGTTTTAGGTAGAG | 487 | Present study |
|  | ND5 | ND55P_for | AACAGCTATCCATTGGTCTTAG | ND55P_rev | CGATGAACAGTTGGAATAGGTTG | 475 | Present study |

**Supplementary Table S5**. Pooling scheme of the PCR products obtained from each mtDNA region.

| **Samples** | **Library ID^1^** | **Region/Gene** | **Volume of**  **PCR reaction (µl)^2^** | **H_2_O (µl)** | **AMPure XP (µl)** |
| --- | --- | --- | --- | --- | --- |
| **EFGH** | 9588 (RL6) | DLOOP | 8.48 |  |  |
|  |  | CO1 | 4.10 |  |  |
|  |  | ND5(-5p) | 7.82 |  |  |
|  |  | Total | 20.40 | 79.60 | 80.00 |
|  | 9589 (RL7) | HV1 | 4.24 |  |  |
|  |  | HV2 | 7.80 |  |  |
|  |  | ND5(-3p) | 11.51 |  |  |
|  |  | Total | 23.55 | 76.45 | 80.00 |
| **I** | 9590 (RL8) | DLOOP | 3.00 |  |  |
|  |  | CO1 | 3.00 |  |  |
|  |  | ND5(-5p) | 16.90 |  |  |
|  |  | Total | 22.90 | 77.10 | 80.00 |
|  | 9591 (RL9) | HV1 | 4.00 |  |  |
|  |  | HV2 | 16.04 |  |  |
|  |  | ND5(-3p) | 4.00 |  |  |
|  |  | Total | 24.04 | 75.96 | 80.00 |
| **I_R_** | 9592 (RL10) | DLOOP | 4.39 |  |  |
|  |  | CO1 | 3.97 |  |  |
|  |  | ND5(-5p) | 6.86 |  |  |
|  |  | Total | 15.22 | 84.78 | 80.00 |
|  | 9593 (RL11) | HV1 | 8.33 |  |  |
|  |  | HV2 | 8.82 |  |  |
|  |  | ND5(-3p) | 5.00 |  |  |
|  |  | Total | 22.15 | 77.85 | 80.00 |

^1^MIDs utilized in each library are in parentheses;

^2^Volume corresponding to 250 ng of amplified DNA.

**Supplementary Table S6**. Number of sequence reads and assembled reads produced from each library.

| **Samples** | **Library ID** | **MID** | **Sequence reads** | **Assembled reads** |
| --- | --- | --- | --- | --- |
| **EGFH** | 9588 | MID 6 | 28570 | 27242 |
| **EGFH** | 9589 | MID 7 | 28765 | 27256 |
| **I** | 9590 | MID 8 | 17877 | 17129 |
| **I** | 9591 | MID 9 | 31142 | 30058 |
| **I_R_** | 9592 | MID 10 | 22832 | 22129 |
| **I_R_** | 9593 | MID 11 | 39919 | 38598 |
| **Total** |  |  | 169105 | 162412 (96.04%) |

**Supplementary Table S7.** Number of aligned reads for each mtDNA region and each library*.*

| **MT Locus** | **Library ID** | | | | | |
| --- | --- | --- | --- | --- | --- | --- |
|  | 9588 | 9589 | 9590 | 9591 | 9592 | 9593 |
| DLOOP | 5031 | - | 3287 | - | 2758 | - |
| ND5(-5P) | 13997 | - | 8220 | - | 12616 | - |
| CO1 | 8214 | - | 5622 | - | 6755 | - |
| HV1 | - | 10069 | - | 11648 | - | 16272 |
| HV2 | - | 2524 | - | 2691 | - | 3968 |
| ND5(-3P) | - | 14663 | - | 15719 | - | 18358 |

**Supplementary Table S8. Human mtDNA haplotypes detected in the Turin Shroud samples.**

| **Sample ID** | **TS Sources** | **GenBank**  **Accession No.** | **MT Locus** | **Sequence Range (from np to np)** | **Haplotype^1,2,3^** | **Potential Contaminants** |
| --- | --- | --- | --- | --- | --- | --- |
| H6XP41B01A4UZC | EFGH | KM655914 | DLOOP | 16303-16569;1-154 | 16519C,73G |  |
| H6XP41B01CDUHR | I | KM655931 | HV2 | 10-424 | 73G,263G | Operator 2 |
| H6XP41B01CEHF7 | EFGH | KM655923 | HV1 | 15986-16425 | 16240G |  |
| H6XP41B01A1XXT | I | KM655924 | HV1 | 15976-16419 | 16234T |  |
| H6XP41B01A8GQS | EFGH | KM655908 | CO1 | 6977-7492 | 7173G,7403G,7404A |  |
| H6XP41B01DKUX8 | I_R_ | KM655909 | CO1 | 6977-7494 | 7403G,7404A |  |
| H6XP41B01CJPBA | I_R_ | KM655930 | HV2 | 10-428 | 263G | Operator 1 |
| H6XP41B01DEI9T | I | KM655929 | HV2 | 10-428 | 263G | Operator 1 |
| H6XP41B01DOHZM | EFGH | KM655927 | HV2 | 20-427 | 263G | Operator 1 |
| H6XP41B01DTAX5 | I_R_ | KM655926 | HV2 | 16-428 | 263G,286T |  |
| H6XP41B01A2DCX | EFGH | KM655932 | HV2 | 10-428 | 263G,363C |  |
| H6XP41B01A6P3E | EFGH | KM655928 | HV2 | 10-426 | 263G,410T |  |
| H6XP41B01CQSWW | I_R_ | KM655881 | ND5(-5P) | 12279-12763 | 12385A,12418T,12427A |  |
| H6XP41B01B3UL0 | I | KM655910 | DLOOP | 16285-16569;1-154 | 16375A,16419A,16519C |  |
| H6XP41B01A8NUY | EFGH | KM655912 | DLOOP | 16281-16569;1-154 | 16519C | Operator 1 |
| H6XP41B01ADT9Q | I | KM655911 | DLOOP | 16281-16569;1-150 | 16519C | Operator 1 |
| H6XP41B01AUT3P | I | KM655903 | CO1 | 6977-7552 | 7316A |  |
| H6XP41B01AI1CL | I_R_ | KM655904 | CO1 | 6977-7494 | 7342T,7402G |  |
| H6XP41B01BTL8I | I | KM655902 | CO1 | 7093-7472 | 7402G |  |
| H6XP41B01A9GL4 | I | KM655891 | ND5(-3P) | 13320-13807 | rCRS | Operators 1 & 3 |
| H6XP41B01BEXJY | I | KM655919 | HV1 | 15986-16421 | rCRS | Operator 1 |
| H6XP41B01COBH3 | EFGH | KM655892 | ND5(-3P) | 13320-13806 | rCRS | Operators 1 & 3 |
| H6XP41B01DSK24 | I_R_ | KM655893 | ND5(-3P) | 13320-13807 | rCRS | Operators 1 & 3 |
| H6XP41B01B1071 | I | KM655889 | ND5(-5P) | 12279-12763 | 12399T,12441C |  |
| H6XP41B01CY9E6 | EFGH | KM655883 | ND5(-5P) | 12279-12763 | 12314C,12372A,12419T |  |
| H6XP41B01AIQYR | EFGH | KM655918 | HV1 | 15980-16425 | 16188T |  |
| H6XP41B01BB6C8 | I_R_ | KM655917 | HV1 | 15976-16425 | 16188T |  |
| H6XP41B01BUK4M | I_R_ | KM655922 | HV1 | 15976-16425 | 16188T |  |
| H6XP41B01BYNPD | I | KM655916 | HV1 | 15980-16425 | 16188T |  |
| H6XP41B01CA5RM | I_R_ | KM655915 | HV1 | 15976-16425 | 16188T |  |
| H6XP41B01BFB78 | I_R_ | KM655921 | HV1 | 15978-16425 | 16179A |  |
| H6XP41B01AID2S | I | KM655890 | ND5(-5P) | 12362-12763 | 12738G | Operator 1 |
| H6XP41B01A9ODT | EFGH | KM655907 | CO1 | 6997-7495 | 7028N^1^,7132T,7146G,7232T,7256T,7316A |  |
| H6XP41B01CV5V3 | I | KM655906 | CO1 | 7051-7532 | 7028N^1^,7394G |  |
| H6XP41B01CCCU7 | I_R_ | KM655886 | ND5(-5P) | 12279-12763 | 12738C | Operator 1 |
| H6XP41B01CIW9S | I | KM655885 | ND5(-5P) | 12279-12763 | 12738C | Operator 1 |
| H6XP41B01DLOZC | EFGH | KM655884 | ND5(-5P) | 12282-12763 | 12738C | Operator 1 |
| H6XP41B01BNWZ3 | I_R_ | KM655913 | DLOOP | 16286-16569;1-154 | 16519C,66T |  |
| H6XP41B01A68KR | I | KM655901 | ND5(-3P) | 13320-13807 | 13753C |  |
| H6XP41B01B7KZJ | EFGH | KM655900 | ND5(-3P) | 13320-13807 | 13753C |  |
| H6XP41B01B7DOS | I | KM655882 | ND5(-5P) | 12279-12763 | 12406A,12738C |  |
| H6XP41B01A4NE1 | I_R_ | KM655896 | ND5(-3P) | 13320-13807 | 13630G,13782T |  |
| H6XP41B01C22EG | EFGH | KM655899 | ND5(-3P) | 13320-13807 | 13751C,13782T |  |
| H6XP41B01CRK64 | I_R_ | KM655898 | ND5(-3P) | 13320-13807 | 13758A,13782T |  |
| H6XP41B01CTG6O | I | KM655897 | ND5(-3P) | 13320-13807 | 13767G,13782T |  |
| H6XP41B01DJQSV | EFGH | KM655905 | CO1 | 6977-7552 | 7232T,7256T,7316A |  |
| H6XP41B01BSDB7 | I_R_ | KM655925 | HV2 | 14-428 | 58C,60T^2^,64T,263G |  |
| H6XP41B01CF148 | I | KM655934 | HV2 | 16-428 | 58C,60T^2^,64T,263G |  |
| H6XP41B01DVQOZ | EFGH | KM655933 | HV2 | 22-428 | 58C,60T^2^,64T,263G |  |
| H6XP41B01B4J2E | I_R_ | KM655920 | HV1 | 15976-16425 | 16051N^1^,16209N^1^,16239N^1^,16352C,16353T |  |
| H6XP41B01BE4DU | I_R_ | KM655895 | ND5(-3P) | 13320-13807 | 13630G,13789C |  |
| H6XP41B01AXGUJ | I | KM655887 | ND5(-5P) | 12279-12763 | 12308G,12372A,12399T,12441C | Operator 2 |
| H6XP41B01C9QLV | I | KM655888 | ND5(-5P) | 12279-12763 | 12308G,12372A,12399T,12441C | Operator 2 |
| H6XP41B01DVXSD | I_R_ | KM655894 | ND5(-3P) | 13320-13807 | 13617N^1^,13630G,13637N^1^ |  |

^1^N indicates that the specified diagnostic mutation falls in a low quality region;

^2^The diagnostic insertion 60+T is likely present as well;

^3^Mutations are relative to the rCRS^17^.
